# A test for plasticity in sperm motility activation in response to osmotic environment in an anuran amphibian

**DOI:** 10.1002/ece3.9387

**Published:** 2022-10-01

**Authors:** Phillip G. Byrne, Zara M. Anastas, Aimee J. Silla

**Affiliations:** ^1^ School of Earth, Atmospheric and Lifesciences University of Wollongong Wollongong New South Wales Australia

**Keywords:** anuran, osmolality, salinity, sperm activation, sperm evolution, sperm motility, sperm velocity

## Abstract

Evolutionary theory predicts that selection will favor phenotypic plasticity in sperm traits that maximize fertilization success in dynamic fertilization environments. In species with external fertilization, osmolality of the fertilization medium is known to play a critical role in activating sperm motility, but evidence for osmotic‐induced sperm plasticity is limited to euryhaline fish and marine invertebrates. Whether this capacity extends to freshwater taxa remains unknown. Here, we provide the first test for plasticity in sperm‐motility activation in response to osmotic environment in an anuran amphibian. Male common eastern froglets (*Crinia signifera*) were acclimated to either low (0 mOsmol kg^−1^) or high (50 mOsmol kg^−1^) environmental osmolality, and using a split‐sample experimental design, sperm were activated across a range of osmolality treatments (0, 25, 50, 75, 100, and 200 ± 2 mOsmol kg^−1^). Unexpectedly, there was no detectable shift in the optimal osmolality for sperm‐motility activation after approximately 13 weeks of acclimation (a period reflecting the duration of the winter breeding season). However, in both the low and high acclimation treatments, the optimal osmolality for sperm‐motility activation mirrored the osmolality at the natural breeding site, indicating a phenotypic match to the local environment. Previously it has been shown that *C. signifera* display among‐population covariation between environmental osmolality and sperm performance. Coupled with this finding, the results of the present study suggest that inter‐population differences reflect genetic divergence and local adaptation. We discuss the need for experimental tests of osmotic‐induced sperm plasticity in more freshwater taxa to better understand the environmental and evolutionary contexts favoring adaptive plasticity in sperm‐motility activation.

## Graphical abstract

Local adaptation to environmental osmolality

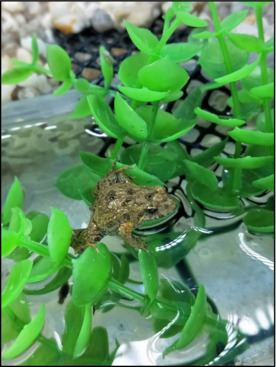

## 1 INTRODUCTION

In species with external fertilization, including many fish, amphibians, and marine invertebrates, gametes released into the environment face extreme spatial and temporal variation in abiotic factors that can influence sperm–egg interactions (Levitan, 2008; Mead & Denny, 1995). Under these conditions, evolutionary theory predicts that selection will favor phenotypic plasticity in gametic traits that maximize fertilization success across a range of environmental conditions. For sperm in particular, changes in various physical and chemical properties of the external environment can influence physiological functioning and fertilization capacity (Alavi & Cosson, 2006; Muto & Kubota, 2009; Ritchie & Marshall, 2013; Simmons et al., 2009). As such, we should expect males to come under strong selection to produce sperm with flexible phenotypes that match the fertilization environment (Jensen et al., 2013). While there is a large body of evidence that the sperm of external fertilizers have evolved to maximize performance in response to specific abiotic environmental factors, such as water salinity, temperature, and pH (Green et al., 2020; Reinhardt et al., 2015), the potential for sperm plasticity in response to variable fertilization environments is yet to be widely investigated (Green, Niemax, et al., 2021; Jensen et al., 2013).

For many external fertilizers, the sperm trait with the strongest influence on fertilization success is sperm motility (Birkhead et al., 2008), typically measured as the proportion of motile sperm released or sperm swimming speed (sperm velocity; Alavi & Cosson, 2006; Au et al., 2002; Dziminski et al., 2010; Evans et al., 2013). While various abiotic factors can influence sperm motility, the key factor controlling the activation of sperm motility is fertilization‐medium osmolality, the total number of dissolved solutes in a solution (Alavi & Cosson, 2006). Prior to ejaculation, sperm are held in isotonic conditions in the testes and are typically immotile (Legendre et al., 2016). It is only after sperm are released into the external environment that sperm motility is activated by a rapid change in osmotic pressure (Alavi & Cosson, 2006; Silla & Byrne, 2019). External fertilizers breed in a range of osmotic environments (spanning freshwater, brackish water, and saltwater), and the osmolality required to activate sperm and initiate motility generally reflects the osmotic environment in which a species has evolved (Alavi & Cosson, 2006; Legendre et al., 2016).

In marine invertebrates and marine teleost fish, a sudden increase in osmolality (hyper‐osmotic relative to seminal plasma) after sperm are released is required for sperm‐motility activation, with the opposite true for freshwater teleosts which require a decrease in osmolality (hypo‐osmotic relative to seminal plasma; Alavi & Cosson, 2006; Legendre et al., 2016). Such changes appear to be controlled by the influx or efflux of specific cations (i.e., K^+^, Ca^+^, Na^+^) and associated changes in osmotic pressure (Alavi & Cosson, 2006). In externally fertilizing amphibians, hypotonic shock experienced by sperm as they contact freshwater promotes an increase in intracellular adenosine monophosphate that in turn triggers protein phosphorylation cascades which are responsible for activating sperm motility (O'Brien et al., 2011). Across taxa, even after hyper‐ or hypo‐ osmotic shock initiates sperm motility activation, sperm remain sensitive to local changes in osmolality and exposure to suboptimal conditions can negatively impact sperm performance (Alavi & Cosson, 2006; Morisawa et al., 1983). Consequently, fertilization success is expected to rapidly decline if sperm are exposed to osmolalities that deviate from a species' optimal osmotic range (Falkenberg et al., 2019; Whiterod & Walker, 2006).

Despite strong evidence that environmental osmolality has influenced the evolution of sperm physiology across species, the extent to which local changes in the osmotic environment might influence sperm activation within species remains poorly understood. Nevertheless, a small number of laboratory‐based acclimation studies spanning six species of euryhaline fish and two marine spawning invertebrates (a tube worm and a sea urchin) have provided some compelling evidence for salinity‐induced phenotypic plasticity in sperm activation. In these species, the optimal osmolality for the activation of sperm motility has been shown to shift and broaden to reflect the osmolality of the acclimation environment (Green, Niemax, et al., 2021; Jensen et al., 2013; Legendre et al., 2016; Linhart et al., 1999; Morita et al., 2004; Palmer & Able, 1987; Taugbol et al., 2017; Tiersch & Yang, 2012). To our knowledge, a lack of plasticity in sperm‐motility activation following acclimation to different osmolalities has only been reported in two fish species: the sand goby (*Pomatoschistus minutus*), a species with a wide geographic distribution in relation to salinity (Svensson et al., 2017), and the euryhaline round goby (*Neogobius melanostomus*; Green, Niemax, et al., 2021), a highly invasive species that might respond to novel osmotic environments through rapid local adaptation (Green et al., 2020). The demonstration of plasticity in sperm‐motility activation in response to osmotic environment in various vertebrate and invertebrate taxa suggests that this adaptive capacity might be widespread. However, because past studies have been restricted to species with an evolutionary history of breeding in saltwater or brackish water, it remains unknown whether plasticity in sperm motility activation has evolved in freshwater taxa.

In freshwater systems, osmolality can change quickly for various reasons, ranging from periods of heavy rainfall causing hypo‐osmotic conditions, to salinization events (linked to ground‐water incursions, sea water incursions, transport of atmospheric sea salt, low water flow, and/or high evaporation rates) causing hyper‐osmotic conditions (Nielsen et al., 2003). Therefore, we should expect that various freshwater species will have a long evolutionary history of males experiencing strong selection to adjust the physiology and functioning of their sperm to suit local osmotic environments. Testing for sperm plasticity in response to osmotic environment in freshwater taxa is needed to better understand the influence of osmotic environment on sperm evolution and the capacity for freshwater species to respond to environmental change. Acquiring this knowledge will be particularly valuable given the global acceleration of freshwater salinization and a growing need to predict impacts of altered osmotic conditions on reproductive outcomes and population viability (Cunillera‐Montcusí et al., 2022).

Anuran amphibians provide an ideal model to test for phenotypic plasticity in sperm‐motility activation in freshwater taxa. Anurans are almost exclusively externally fertilizing, and for several decades, it has been known that sperm motility is activated in freshwater of very low osmolality (0–25 mOsmol kg^−1^; Silla & Byrne, 2019). However, with a surge in research focused on developing artificial fertilization protocols for threatened species, evidence has emerged that sperm motility may be activated in media up to and beyond 100 mOsmol kg^−1^(Costanzo et al., 1998; Silla, 2013; Silla et al., 2017). Many anurans are widely distributed and have been observed breeding in a diversity of osmotic environments (Albecker & McCoy, 2017; Hopkins & Brodie Jr., 2015). Differences in sperm performance have also been reported between populations (Byrne et al., 2015; Hettyey & Roberts, 2006; Rudin‐Bitterli et al., 2020), with recent evidence that extensive geographical variation in sperm motility is associated with local patterns of precipitation (Rudin‐Bitterli et al., 2020). Together, these observations suggest that local osmotic environments may have played an important role in the evolution of anuran sperm physiology and functioning.

As a first step towards investigating this possibility, a previous study tested for among‐population co‐variation between sperm performance and environmental osmolality in the Australian common eastern froglet *Crinia signifera* (Byrne et al., 2015), a widely distributed species that has been observed breeding in brackish habitats (Hopkins et al., 2020). It was discovered that males from populations with higher environmental osmolality retained more motile sperm when activated at higher osmolalities (Byrne et al., 2015). While this finding might reflect phenotypic plasticity in sperm‐motility activation, it might also be explained by genetic divergence in sperm functioning and local adaptation. To distinguish between these possibilities, the aim of the present study was to use an acclimation experiment to test whether environmental osmolality can induce plastic responses in sperm‐motility activation in *C. signifera*. Based on the assumption that sperm will adapt to their prevailing osmotic environment, we predicted that frogs acclimated to higher osmolality would produce sperm that performed better at higher osmolalities in vitro.

## 2 MATERIALS AND METHODS

### 2.1 Ethical note

All procedures described in this manuscript were conducted following evaluation and approval by the University of Wollongong's Animal Ethics Committee (approval number AE21/12) in accordance with the Australian Code for the Care and Use of Animals for Scientific Purposes 2013. Procedures were also authorized by New South Wales National Parks & Wildlife Service Office of Environment and Heritage (SL102528).

### 2.2 Study species

*Crinia signifera* is a small (snout‐vent length = 15–30 mm) ground‐dwelling Australian frog from the family Myobatrachidae. Breeding is aquatic and typically occurs in ephemeral water bodies over a 12‐ to13‐week period spanning late autumn and winter (Anstis, 2013). During the breeding season, males aggregate in shallow water and use advertisement calls to attract females. During mating, males grasp females around the waist (inguinal amplexus) and females lay an average of 216 eggs (range 125–394; Anstis, 2013). The eggs are encapsulated in two distinct jelly layers, formed as the eggs pass through the oviduct prior to deposition in shallow water near a males' advertisement site (Anstis, 2013). During egg deposition, amplectant males release flagellated sperm (Byrne et al., 2003), and fertilization occurs at the site of gamete release.

### 2.3 Study site and frog collection

All frogs were collected from a single population located adjacent to the Ecological Research Centre (ERC) at the University of Wollongong (34.4048° S, 150.8717° E). This site was chosen as it was a defined breeding site characterized by shallow ephemeral water bodies surrounded by grasses and riparian vegetation. Moreover, osmolality at the site was expected to be variable due to the site's close proximity to the coast (approximately 2 km). In order to gain an understanding of variation in osmolality at the breeding site, osmolality was measured at multiple point locations weekly for 11 weeks from the May 27, to the August 5, 2021. Each week 3 to 5 individual water samples were collected, and the osmolality of each sample was measured using a freezing‐point depression osmometer (Osmomat 3000; GONOTEC, Germany). Osmolality ranged between 7.33 and 10.17 mOsmol kg^−1^ (mean = 8.68 ± 0.33 mOsmol kg^−1^). Frogs were collected during the winter of 2021, on June 4 and 20, after periods of light rainfall when calling activity increased. Collections were performed at night, between 17:30 and 22:30. On the June 4, 13 adult males were collected, and on the June 20, an additional 3 males were collected. The frogs were caught by hand after tracking their vocalizations and immediately after capture frogs were placed in sealed plastic bags. At the end of each collection period, frogs were transported to an experimental constant temperature room in the Ecological Research Centre at the University of Wollongong.

### 2.4 Animal husbandry

**FIGURE 1:**
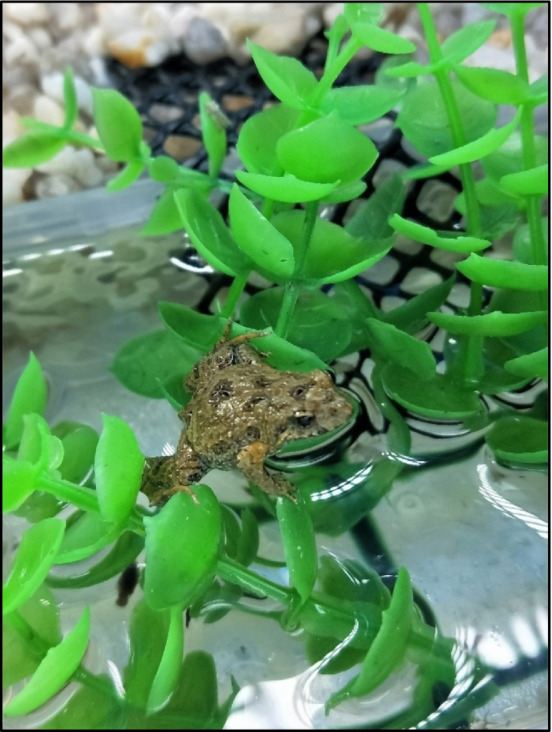
Adult male common eastern froglet, *Crinia signifera*, shown positioned in a water dish containing the acclimation treatment solution. Image courtesy of Zara Anastas.

Frogs were housed in individual ventilated terraria (27 cm × 17 cm × 16 cm). Each terraria contained two cups of aquarium gravel, a section of PVC piping (3 cm in diameter, 5 cm long), and a 300‐ml rectangular plastic water dish (10 cm × 6 cm × 5 cm) containing the acclimation treatment solution (Figure 1). Equally sized pieces of plastic aquarium plant and gutter guard were placed in the water dish to allow the frogs to move in and out of the water. Each terrarium had 8 holes (~0.5 cm in diameter) along the underside to allow for drainage. Terrariums were kept on two shelves (8 terrariums on each of the upper and lower shelves) with terrariums placed on the shelves in alternating treatment order. One side of each terrarium was covered in black dampcourse (16 cm × 9 cm) to prevent visual contact between males and the possibility that male–male interactions influenced sperm production or performance (Lüpold et al., 2017; Magris, 2021).

Frogs (*n* = 16) were kept in a constant temperature room maintained at 18°C, simulating the average maximum temperature for the study site in July (data obtained from Bellambi weather station from 1997–2021, Bureau of Meterology, 2021). The room was maintained on a 10: 14 hr light dark cycle simulating natural lighting conditions during winter in the Illawarra region. Broad spectrum UV light was supplied by Reptisun 36″ high output fluorescent strip bulbs (Pet Pacific Pty Ltd) suspended approximately 20 cm above the terrariums. Frogs were fed an ad libitum diet of hatchling (first instar) live crickets (*Acheta domestica*) twice a week. To prevent metabolic bone diseases caused by a calcium and/or vitamin D_3_ deficient diet, crickets were dusted with approximately 200 mg of calcium powder with added D_3_ (Repti Calcium; Zoo Med) immediately prior to feeding (Ferrie et al., 2014). Frogs remained in captivity for a total of 14 weeks prior to sperm collection.

Terrariums were flushed with R.O. water (Sartorius Stedim biotech, Australia) once a week (~1000 ml) to remove excrement and uneaten food, and all water dishes were completely changed immediately afterwards to ensure that the osmolality of the water was unaltered. Additionally, water in the dishes was changed three times a week (using a 50‐ml syringe to siphon water, excrement, and excess food) and was replaced by completely filling the water dish with water of the appropriate treatment osmolality (see below). On days that a complete water change did not occur, water dishes were topped up with treatment water.

### 2.5 Experimental design

**FIGURE 2:**
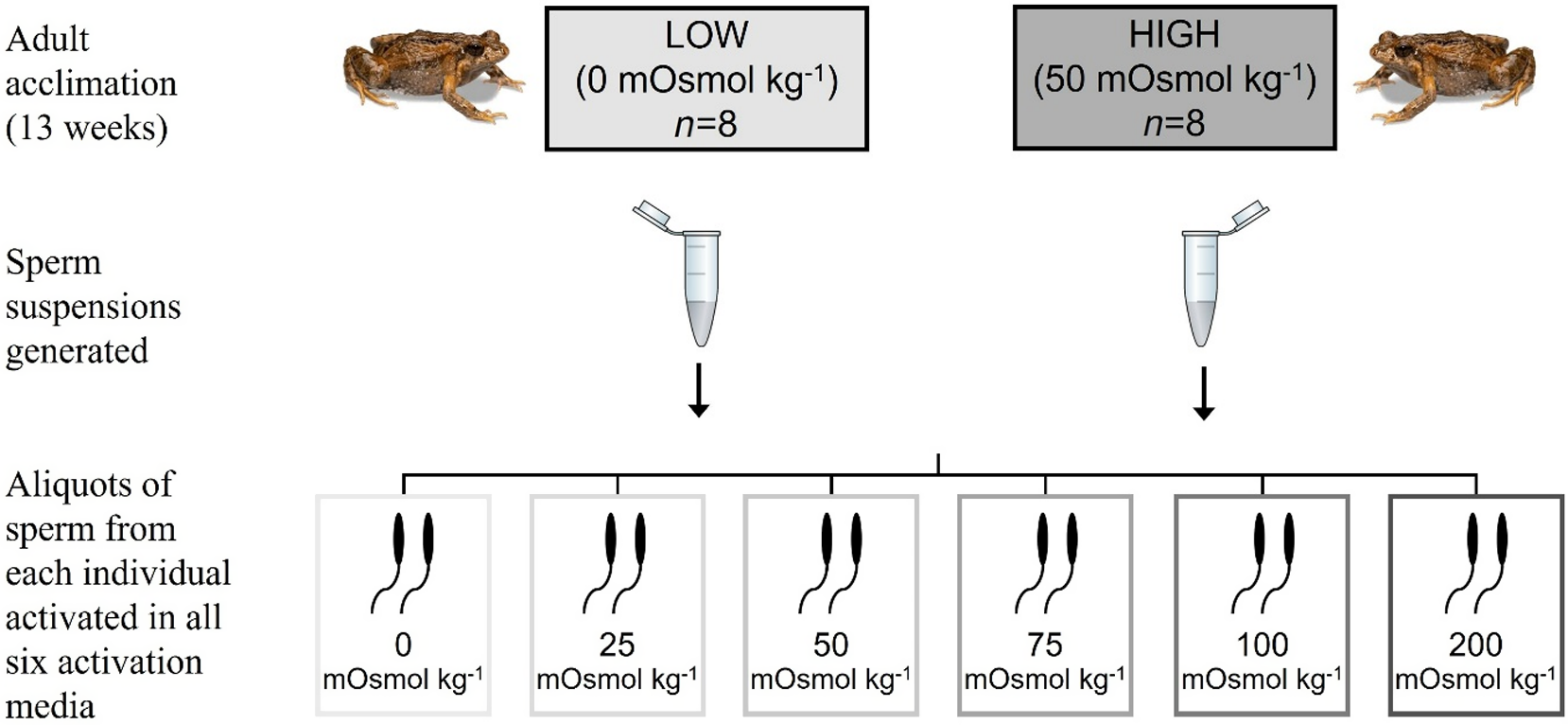
Diagram of 2‐treatment split‐sample experimental design. Frogs were acclimated for 13 weeks to water at low osmolality (0 mOsmol kg^−1^) or high osmolality (50 mOsmol kg^−1^), and sperm samples were collected from each. Each sperm suspension (*n* = 8 per treatment) split into six aliquots and activated in media of experimental osmolalities: 0, 25, 50, 70, 100 or 200 mOsmol kg^−1^.

Males (*n* = 16) were randomly allocated to one of two acclimation treatments, low (0 mOsmo lkg^−1^) or high (50 mOsmol kg^−1^) osmolality, with 8 frogs per treatment (Figure 2). These acclimation treatment osmolalities were chosen to reflect the osmolality of freshwater and the maximum breeding‐site osmolality reported in two previous studies (Byrne et al., 2015; Hopkins et al., 2020). Water osmolality was adjusted incrementally over a period of 4 days to avoid causing physiological stress. After frogs were first allocated to terrariums, their water dishes were filled with 300 ml of water with an osmolality of 10 mOsmol kg^−1^ (approximately equal to the collection site osmolality). The osmolality of this solution and all subsequent solutions were prepared by diluting simplified amphibian ringer (SAR; composition [in mM]: NaCl 113; CaCl_2_ 1; KCl 2; NaHCO_3_ 3.6; 250 mOsmol kg^−1^) in distilled water to the desired osmolality, and the osmolality of each was confirmed at least twice by a freezing‐point depression osmometer (Osmomat 3000; GONOTEC, Germany). On the fourth day of captivity, a 4‐day adjustment period began (7 day adjustment total). For the low treatment, water remained at 10 mOsmol kg^−1^ for 3 days before being replaced with the 0 mOsmol kg^−1^ treatment solution. For the high treatment, osmolality was increased by 10 mOsmol kg^−1^ each day and reached 50 mOsmol kg^−1^ on the fourth day. Throughout this adjustment period, each water dish was completely emptied and replaced using a syringe. Once each frog's water reached the acclimation treatment osmolality, the 13‐week experimental period began, with water osmolality maintained at either 0 mOsmol kg^−1^ (range = 0–1 mOsmol kg^−1^) or 50 mOsmol kg^−1^ (range = 48–53 mOsmol kg^−1^). An acclimation period of 13 weeks was chosen to encompass the entire winter‐breeding season of the study population and ensure that the data were ecologically relevant.

To determine the effect of acclimation osmolality on sperm motility, sperm were activated across a range of osmolalities using a split‐sample experimental design (Figure 2). Sperm suspensions sampled from each frog (*n* = 8 per treatment) were aliquoted and placed in six different activation media: 0, 25, 50, 75, 100, and 200 ± 2 mOsmol kg^−1^ (Figure 2). Activation mediums were prepared in the same way as the acclimation treatment solutions, and the osmolality of each was confirmed using a freezing‐point depression osmometer. This experimental design follows that used in previous studies investigating anuran sperm activation at different fertilization medium osmolalities (Byrne et al., 2015; Silla, 2013; Silla et al., 2017).

### 2.6 Preparation of sperm suspensions

After the acclimation period, sperm were sampled on the September 9 and 10 (for the frogs collected on June 4) and September 24 (for the frogs collected on June 20). Accordingly, the exact acclimation period for frogs in each treatment group was 90 to 91 days. Each frog was weighed to the nearest 0.001 grams (range = 0.876 to 1.593 g, mean ± SEM = 1.10 ± 0.18 g, *n* = 16) and photographed to measure snout‐vent length (mm) using the program ImageJ. Snout‐vent length was measured from the apex of the head to the cloaca (range = 19.87 to 25.86 mm, mean ± SEM = 22.70 ± 0.17 mm). Immediately after frogs were weighed and photographed, they were euthanized via pithing (brain destruction) and both testes were removed by dissection and weighed to the nearest 0.0001 grams. Testes mass ranged from 0.0016 to 0.0049 g (mean ± SEM = 0.0023 ± 0.00025 g). The testes of each individual were then macerated in 100 μl chilled simplified amphibian ringer (SAR; composition [in mM]: NaCl 113; CaCl_2_ 1; KCl 2; NaHCO_3_ 3.6; 250 mOsmol kg^−1^) and stored on ice in 1.5 ml Eppendorf tubes. For each suspension, sperm concentration was quantified using a Neubauer improved hemocytometer (Bright Line; Optik Labour; exact depth 0.1 mm). A 2 μl homogenized subsample of sperm suspension was diluted in 18 μl of SAR (1:10 dilution), homogenized, and then pipetted into the hemocytometer chamber. The number of spermatozoa present in five quadrats was recorded. The dilution and counting protocol was then repeated twice per suspension and averaged to calculate total sperm concentration. Sperm suspensions (*n* = 16) ranged in concentration from 8.50 to 55.00 × 10^7^ spermatozoa L^−1^ (mean ± SEM = 19.75 ± 1.20 × 10^7^ spermatozoa L^−1^).

### 2.7 Sperm motility activation and assessment

Sperm motility was activated in the six activation media (0, 25, 50, 75, 100, and 200 ± 2 mOsmol kg^−1^) according to methods described previously (Silla et al., 2017). Sperm suspensions were homogenized and six discrete 2 μl subsamples were removed and diluted in 18 μl of each of the six chilled activation media (1: 10 dilution). The order in which each activation treatment was applied was randomized for each sperm suspension using a random‐number generator. Sperm motility variables were assessed following a 5‐min activation period, including a 2 min settlement, where the suspension was pipetted into a hemocytometer chamber (exact depth 0.1 mm) and placed on the microscope stage to allow fluid to settle prior to analysis. A fluid settlement period was necessary to ensure accurate sperm motility readings that were unaltered by fluid dynamics. The 5 min activation period allowed sufficient time for the initiation of sperm motility and for samples to reach room temperature. This process was repeated for each of the six activation mediums, with the order in which each activation treatment was applied randomized for each sperm suspension using a random‐number generator.

Sperm motility variables, percent motility (% of sperm recorded as motile), sperm VAP (average velocity of the smoothed sperm path; μms^−1^), and sperm VCL (average velocity of the curvilinear sperm path; μms^−1^) were measured using a computer‐assisted sperm analysis (CASA) system (CEROS Version 12; Hamilton Thorne). The CASA system used throughout the experiment was set to capture 30 frames per second for 30 frames, cell detection was set to detect a cell size of 5 pixels, with a minimum cell detection contrast of 15, a static cell size of 20 pixels, and a static cell intensity of 80. VAP cut‐off and was 5 μms^−1^ and minimum motility value was 0 μms^−1^, so that slow moving sperm (those with VAP < 5 μms^−1^) contributed to motility measures. Immotile sperm (VAP = 0 μms^−1^) did not contribute to sperm velocity measures. These settings optimized the ability for spermatozoa to be accurately detected and differentiated from cellular debris and ensured that all motile cells were recorded. Each sperm performance assessment was repeated five times per subsample and measured averaged, with each assessment captured for a different position of the slide. The total time taken to generate the five replicate measurements, including the time taken to change the position of the slide, was approximately 60 s. Activation of sperm suspensions and the measurement of sperm motility variables were conducted in a constant temperature room set to at a stable bench temperature of 18°C (mean ± SEM = 18.62 ± 0.082°C; range 17.9–19.5°C).

### 2.8 Statistical analysis

Linear mixed‐effects (LME) models were used to examine the relationship between acclimation treatment, activation medium osmolality, and the interaction between acclimation treatment and activation medium osmolality on sperm performance. Three separate models were run with percent motile sperm, sperm VAP, and sperm VCL as the dependent response variables (calculated as an average from five technical replicates). In each model, acclimation treatment (low, high), activation medium osmolality (0, 25, 50, 75, 100, and 200 mOsmol kg^−1^), and the interaction between acclimation treatment and activation medium osmolality were treated as fixed effects. Frog ID was included as a random effect to account for repeated sampling of the same individuals across the experiment. In models where significant effects were detected, post hoc comparisons were made using Tukey–Kramer Honestly Significant Difference (HSD) tests. Prior to analysis, assumptions of normal distribution for percent motile sperm, sperm VAP, and sperm VCL variables were tested using Shapiro–Wilk tests. Because data were not normally distributed, percent motile sperm data were transformed using an arcsine square root transformation (sin^−1^[√*x*]), and sperm VAP and sperm VCL were transformed using a natural log transformation. Prior to running the main models, associations between sperm performance variables and frog mass (g), frog snout‐vent length (mm) and sperm concentration (×10^7^) were examined with simple regression analyses to determine if they should to be included as co‐variates. No significant correlations were found between any of the variables, so they were excluded from the models. All statistical analyses were performed using the JMP 16.0 software package (SAS Institute Inc.).

## 3 RESULTS

### 3.1 Effect of acclimation treatment on sperm performance

There was no effect of acclimation treatment on any of the sperm performance parameters (linear mixed effect model [LME]: sperm motility; *F*_1,1_ = 0.3491, *p* = .5640; sperm VAP; *F*_1,1_ = 3.6494, *p* = .0767; sperm VCL; *F*_1,1_ = 1.8055, *p* = .2004), and no significant interaction between acclimation treatment and activation medium osmolality (LME: sperm motility; *F*_1,5_ = 2.2316, *p* = .0606; sperm VAP; *F*_1,5_ = 0.5615, *p* = .7291; sperm VCL; *F*_1,5_ = 0.9450, *p* = .4576).

### 3.2 Effect of activation‐medium osmolality on sperm performance

#### 3.2.1 Sperm motility

**FIGURE 3:**
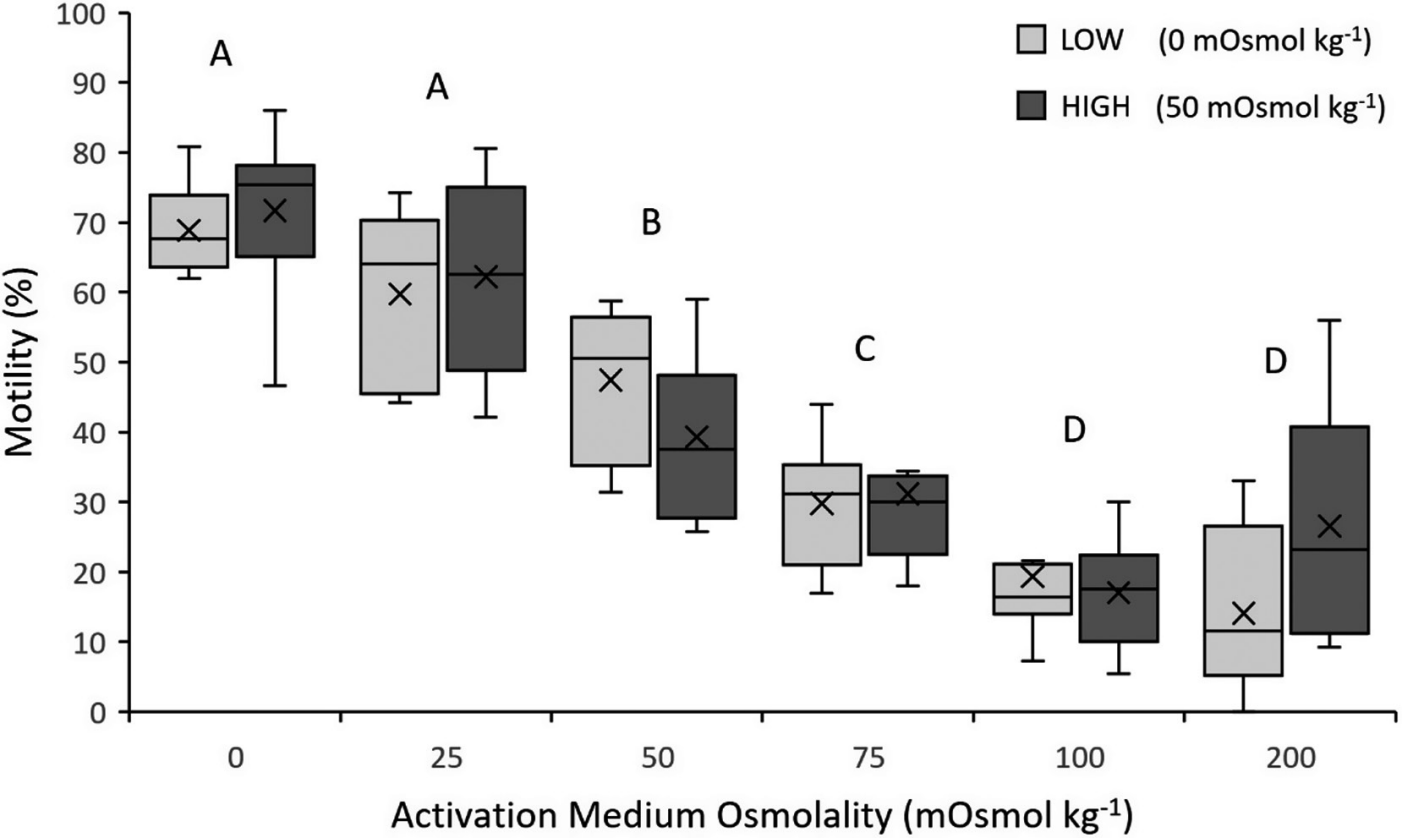
Percent motile sperm of *Crinia signifera* in response to six activation medium osmolalities following acclimation at low or high treatment osmolality. Data shown are untransformed mean ± standard error mean (SEM). Letters displayed are the result of a Tukey–Kramer HSD post hoc test on arcsine transformed data run across both acclimation treatments. Activation medium osmolalities that share a letter are not significantly different from each other (*p* > .05).

There was a significant effect of activation medium osmolality on percent sperm motility (LME: *F*_1,5_ = 65.0311, *p* < .0001). Average sperm motility was highest at 0 mOsmol kg^−1^ (70.28%) and sperm motility decreased as osmolality increased, with the lowest average sperm motility at 100 mOsmol kg^−1^ (18.18%; Figure 3; for mean values for each acclimation treatment, see Table S1). Post hoc Tukey–Kramer HSD tests indicated that sperm motility significantly decreased at 50, 75, and 100 mOsmol kg^−1^ (Figure 3).

#### 3.2.2 Sperm velocity

**FIGURE 4:**
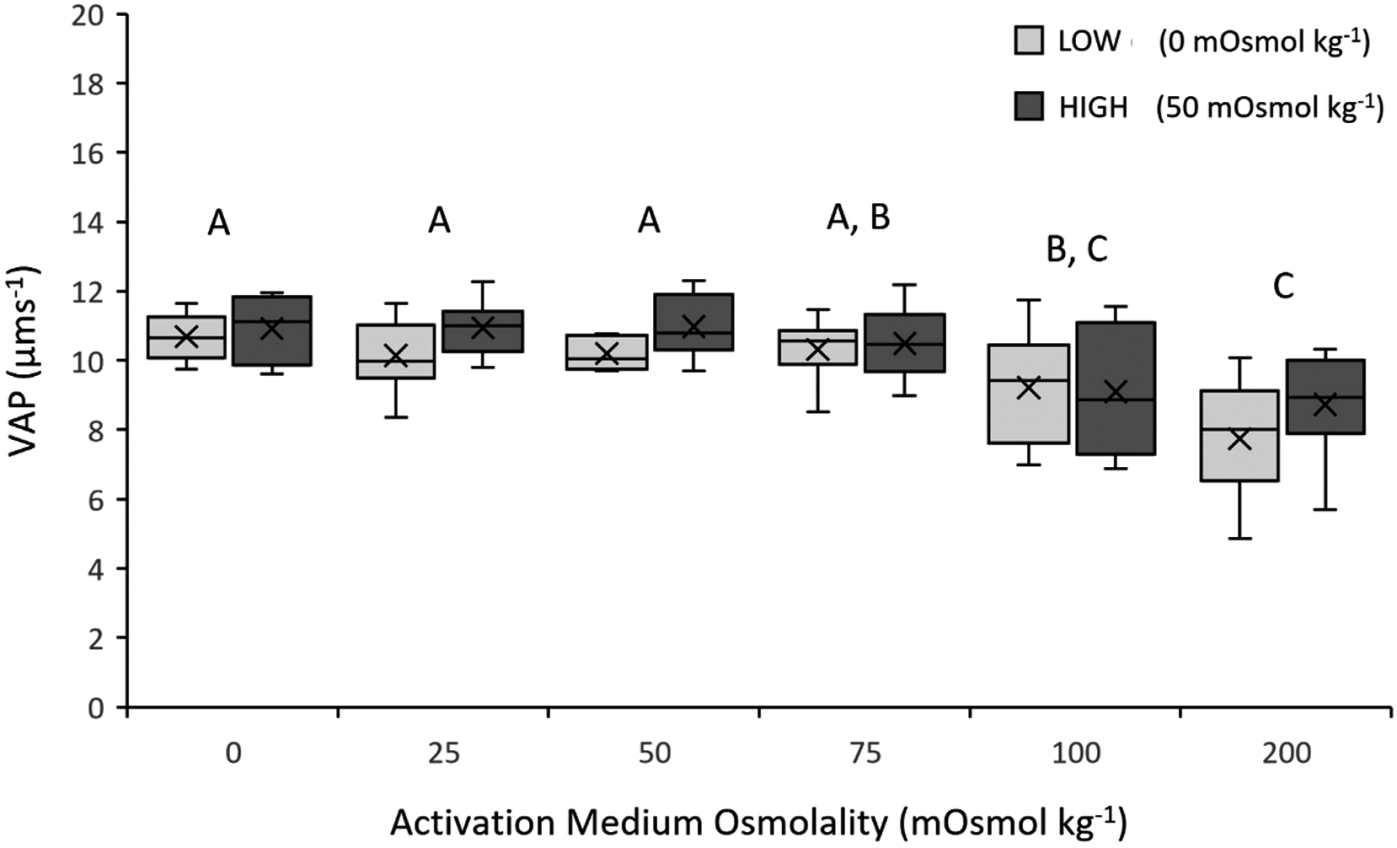
Sperm velocity (VAP, average velocity of the smoothed sperm path; μms^−1^) of *Crinia signifera* in response to six activation medium osmolalities following acclimation at low or high treatment osmolality. Data shown are untransformed mean ± standard error mean (SEM). Letters displayed are the result of a Tukey–Kramer HSD post hoc test on arcsine transformed data run across both acclimation treatments. Activation medium osmolalities that share a letter are not significantly different from each other (*p* > .05).

There was a significant effect of activation medium osmolality on sperm VAP (LME: *F*_1,5_ = 11.0921, *p* < .0001). Average sperm VAP was highest at 0 mOsmol kg^−1^ (10.80 μms^−1^) and was lowest at 200 mOsmol kg^−1^ (8.27 μms^−1^; Figure 4; for mean values for each acclimation treatment; see Table S1). Post hoc Tukey–Kramer HSD tests indicated that sperm VAP significantly decreased at 100 mOsmol kg^−1^, with no significant differences between 0, 25, 50, and 75 mOsmol kg^−1^ (Figure 4).

**FIGURE 5:**
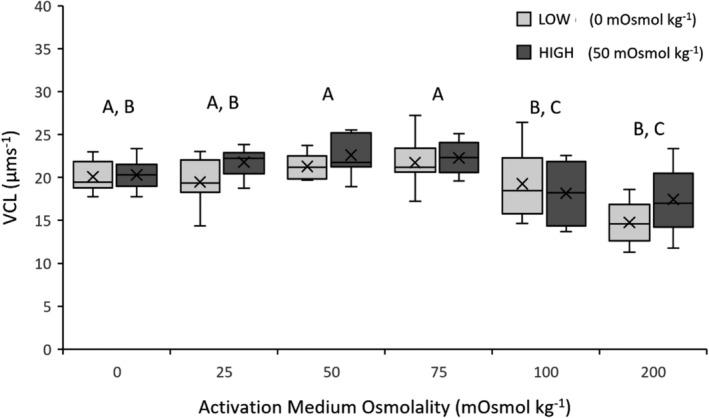
Sperm velocity (VCL, average velocity of the curvilinear sperm path; μms^−1^) of *Crinia signifera* in response to six activation medium osmolalities following acclimation at low or high treatment osmolality. Data shown are untransformed mean ± standard error mean (SEM). Letters displayed are the result of a Tukey–Kramer HSD post hoc test on arcsine transformed data run across both acclimation treatments. Activation medium osmolalities that share a letter are not significantly different from each other (*p* > .05).

There was also a significant effect of activation medium osmolality on sperm VCL (LME: *F*_1,5_ = 10.9179, *p* < .0001). Average sperm VCL was highest at 75 mOsmol kg^−1^ (22.01 μms^−1^) and was lowest at 200 mOsmol kg^−1^ (16.49 μms^−1^; Figure 5; for mean values for each acclimation treatment; see Appendix S1). Post hoc Tukey–Kramer HSD tests indicated that sperm VAP significantly decreased at 100 mOsmol kg^−1^, with no significant differences between 0, 25, 50, and 75 mOsmol kg^−1^ (Figure 5).

## 4 DISCUSSION

Whether freshwater taxa have the capacity for plasticity in sperm‐motility activation remains unknown. The present study aimed to test for osmotic‐induced phenotypic plasticity in sperm‐motility activation in adult male common eastern froglets (*C. signifera*). There was no detectable shift in the optimal osmolality for sperm‐motility activation following acclimation, providing no evidence for sperm plasticity in response to an altered osmotic environment. However, there was evidence that the osmolality of the activation medium had a significant effect on sperm motility and velocity. Optimal sperm performance occurred between 0 and 25 mOsmol kg^−1^ for percentage sperm motility and between 0 and 75 mOsmol kg^−1^ for sperm velocity. These results indicate that the proportion of sperm activated was optimal at very low osmolalities, but that the swimming speed of sperm that were activated was statistically similar across a broad range of osmolalities post motility activation. Moreover, percentage sperm motility (reflecting successful activation) was optimal in the activation medium (0 mOsmol kg^−1^) closest in osmolality to the breeding‐site osmolality (mean _=_ 8.68 ± 0.33 mOsmol kg^−1^), indicating a possible phenotypic match in sperm functioning to the local osmotic environment.

The lack of evidence for plasticity in *C. signifera* sperm‐motility activation following acclimation was unexpected because this capacity has been reported for various externally fertilizing euryhaline fish species that breed across different osmotic environments (Jensen et al., 2013; Legendre et al., 2016; Linhart et al., 1999; Morita et al., 2004; Taugbol et al., 2017; Tiersch & Yang, 2012). Several aspects of our acclimation method may have prevented us from detecting sperm plasticity. First, the acclimation period we imposed may have been too short for a plastic response to manifest. This seems unlikely, however, because acclimation encompassed a period of peak breeding for the study population and lasted for 13 weeks. The aforementioned acclimation studies in fish detected plastic sperm responses within this timeframe. In the most extreme case, a study using three‐spine stickleback (*Gasterosteus aculeatus*) demonstrated changes in just 2 days (Taugbol et al., 2017). However, three‐spine stickleback may be unusual in that spermatogenesis is completed prior to the breeding season, with sperm plasticity facilitated by changes to the osmolality of the seminal plasma surrounding mature sperm cells within the testes. For the other euryhaline species tested, changes to sperm‐motility activation were reported after <8 weeks of acclimation, with some species showing plastic responses after just 2 weeks (Legendre et al., 2016; Linhart et al., 1999; Morita et al., 2004; Palmer & Able, 1987; Tiersch & Yang, 2012). Emerging evidence indicates that these plastic responses are controlled by changes in the sperm motility apparatus (mediated by the differential expression and redistribution of transmembrane water channels mediated by protein phosphorylation pathways) during the process of spermatogenesis (Chauvigne et al., 2013; Morita et al., 2004). The spermatogenic cycle for *C. signifera* is yet to be studied, though it is well established that breeding occurs throughout the year. For anurans with this reproductive strategy, spermatogenesis is expected to be continuous (Ferreira et al., 2008), with a cycle of fresh sperm production (encompassing the full spermatogenic process) expected to occur in under 40 days (Jamieson, 2003; Segatelli et al., 2009). Assuming this is the case for *C. signifera*, the period of acclimation imposed (13 weeks) should have provided sufficient time for a complete cycle of sperm production and shifts in sperm performance.

An alternative explanation for a lack of plasticity is that the high osmolality treatment imposed was not extreme enough to induce a shift in sperm‐motility activation. This seems unlikely, however, because we based our high acclimation treatment (50 mOsmol kg^−1^) on two previous studies spanning various sites located in different parts of the species range. These studies reported that breeding‐site osmolality ranged from 15.5 to 30.2 mOsmol kg^−1^ (Byrne et al., 2015) and from 10 to 50 mOsmol kg^−1^ (Hopkins et al., 2020). In the present study, the mean breeding site osmolality was approximately 9 mOsmol kg^−1^, and the highest single reading over the 11 week sampling period was less than 10.2 mOsmol kg^−1^. Based on these data, there is reason to think that the high acclimation treatment was towards the upper end of osmolality levels across most natural breeding sites. In support of this notion, sperm performance in the present study significantly decreased beyond 50 mOsmol kg^−1^, mirroring the results of a previous study (Byrne et al., 2015). Interestingly, however, in both these studies, a proportion of sperm (approximately 10%–26%) were still motile (though with significantly reduced velocity) in the 100 and 200 mOsmol kg^−1^ treatment osmolalities. The sperm of most frog species is thought to become immotile in osmolalities above 75 mOsmol kg^−1^, with only three examples exceeding this limit (Costanzo et al., 1998; Silla, 2013; Silla et al., 2017). As such, *C. signifera* sperm appear to have a broad osmotic tolerance. The upper osmotic tolerance of adult male *C. signifera* is yet to be tested, though chronic exposure experiments have demonstrated that larvae can complete metamorphosis in brackish water up to 5.0 ppt (approximately 150 mOsmol kg^−1^; Hopkins et al., 2020). Based on this knowledge, there may be value in testing the upper salinity tolerance for male *C. signifera* and repeating our study after acclimating individuals to more challenging osmotic conditions.

Assuming the acclimation method used was appropriate, the present study provides no evidence for phenotypic plasticity in sperm functioning. This may reflect constraints on the evolution of plasticity due to costs of producing different phenotypes (i.e., fitness tradeoffs from investment in different traits), or costs of plasticity (i.e., the reduction to fitness resulting from investment in producing the genetic machinery needed to regulate phenotypic responses; Snell‐Rood & Ehlman, 2021). Alternatively, there may have been no selection for sperm plasticity in the study population. Plasticity is predicted to evolve in response to contrasting selection pressures associated with heterogenous environments and frequent shifts in phenotypic optima (Pfennig, 2021). We saw no evidence for such conditions. The osmolality at the breeding site was low and remarkably stable, despite the study occurring after an extended period of low rainfall, and there being no major rain events during the monitoring period. We suspect that this relates to the breeding site being located at the bottom of an escarpment and connected to a drainage line that maintains a regular supply of freshwater. Under these stable conditions, more specialized phenotypes are likely to be favored. However, the situation may be very different for *C. signifera* populations where breeding takes place in brackish environments, such as coastal swamps (Hopkins et al., 2020). Moving forward, deeper insights into the potential for plasticity in sperm motility activation in *C. signifera* might come from testing frogs adapted to more extreme and variable osmotic environments.

In the absence of any evidence for sperm plasticity, our findings support the notion of adaptive divergence of sperm‐motility activation in response to environmental osmolality. In our previous study, we reported sperm‐phenotype matching to osmotic environment, and this was also evident in the present study with the optimal osmolality (0 mOsmol kg^−1^) being similar to the breeding site osmolality (approximately 9 mOsmol kg^−1^) irrespective of whether frogs were acclimated to a low or high osmolality. This pattern suggests that frogs in the study population might function better under freshwater conditions that are similar to local osmotic conditions. Of note, the osmolality of the study site is also likely to have been higher than normal due to the study taking place during an extended dry period. Importantly, this result is in line with a recent study of salt tolerance in *C. signifiera* larvae, which reported that the degree of salinity tolerance following acute and chronic exposure varies between populations (Hopkins et al., 2020). Therefore, it seems that directional selection imposed by the osmotic environment has led to divergence in physiological functioning over multiple life stages. Theoretically, strong genetic divergence between populations and local adaptation is expected when directional selection operates on nonplastic traits that do not conform to local environmental conditions in genetically isolated populations. Past studies of *C. signifera* population dynamics have revealed strong site fidelity (evidenced by high recapture rates) as well as high mortality rates (caused by intense predation on active frogs; Lemckert & Shine, 1993). Ecological features restrict the rate of migration and gene flow. Concordant with these observations, molecular studies have shown that *C. signifera* is characterized by very high levels of genetic structuring throughout its range (Read et al., 2001; Symula et al., 2008), indicating high levels of genetic divergence and potential for local adaptation.

With the objective of conclusively demonstrating local adaptation in sperm motility activation in *C. signifera*, our study should be repeated using mean local osmolality as a test condition. Furthermore, two additional pieces of evidence will be required. First, it will be necessary to use reciprocal transplant and/or common garden experiments to compare the relative sperm performance of local versus foreign genotypes in local versus foreign fertilization environments. A genotype (population) by environment (activation osmolality) interaction will be indicative of local adaptation (Albecker et al., 2021; Blanquart et al., 2013). Second, it will be necessary to demonstrate that sperm activation has a genetic basis and that divergence in genotypes and phenotypes represents directional selection rather than genetic drift (Endler, 1986). To this end, advances in genomics are permitting genome‐wide scans and assessment of patterns of gene expression that in combination with common garden experiments, breeding experiments and quantitative genetic approaches are helping to identify candidate loci underpinning genetic adaptation and elucidate the genetic architecture of traits targeted by selection (Albecker et al., 2021). Of note, a recent study in the euryhaline round goby (*Neogobius melanostomus*) linked sperm performance in different salinities to population‐genomic patterns in Northern Europe (Green, Apostolou, et al., 2021). A similar approach might help to identify locally adapted *C. signifera* populations. It should also be highlighted that epigenetic inheritance might contribute to among‐population variation in sperm function, justifying investigation into trans‐generational patterns of gene expression (Rodríguez‐Romero et al., 2016).

Our study is the first to test whether frogs can plastically adjust their sperm physiology to match environmental osmolality following osmotic acclimation. As such, there is an urgent need for additional empirical research in this vertebrate order. There are over 4000 described anuran species, affording a wealth of opportunities to test for sperm plasticity. The vast majority of anurans use external fertilization and the group has the highest diversity of reproductive modes of any tetrapod group, with the use of breeding habitats ranging from fully aquatic to fully terrestrial, exposing different species to a range of osmotic environments. A review of saline habitat utilization by amphibians has reported that at least 124 species of anurans globally use saline habitats, including coastal tidal pools, salt marshes, and desert pools (Hopkins & Brodie Jr., 2015). Salinity tolerance has been tested in a diversity of species, with evidence for tolerance to osmotic stress demonstrated for eggs, larvae, and adults (Albecker & McCoy, 2017; Hopkins & Brodie Jr., 2015). There are also some remarkable examples of adaptation to extreme osmotic environments, such as the crab‐eating frog of southeast Asia (*Fejervarya cancrivora*), which can move between freshwater and seawater by rapidly altering levels of urea in body tissue to alter ion transfer and avoid cutaneous water loss and dehydration (Wright et al., 2004). While such physiological capabilities suggest a surprisingly high adaptability of anurans to saline environments, the potential for adaptive responses by sperm has not been considered. This is surprising because interpopulation variation in sperm motility has been reported for several species (Hettyey & Roberts, 2006; Rudin‐Bitterli et al., 2020). Anuran sperm performance is known to have a direct impact on fertilization success and male fitness, with expected flow on effects for population viability (Dziminski et al., 2010). Consequently, tests for sperm plasticity or local adaption in response to variable osmotic environments will improve our understanding of amphibian reproductive biology and evolutionary ecology.

An improved understanding on anuran sperm functioning and adaptive potential also stands to benefit amphibian management and conservation. Anurans are the most threatened vertebrate group, and salinization of freshwater habitats resulting from various anthropogenic activities (agriculture, road salting, and mining) is a key threatening process. Remarkably, salt treatment of ponds has also recently been proposed to combat a fungal pathogen (chytrid fungus) that is decimating amphibian populations globally (Stockwell et al., 2012). Initial studies have shown that elevating ponds to osmolalities of approximately 85 mOsmol kg^−1^ can eliminate the fungus, but this osmolality exceeds the upper limit (50 mOsmol kg^−1^) for sperm performance for most anurans studied to date. If anuran sperm lack phenotypic plasticity in response to osmotic change, salinization as a management strategy has the potential to compromise fertilization success and reduce population viability for many species. Local adaptation in sperm functioning could also pose problems for other management practices. For example, the practice of augmenting declining populations with frogs to increase genetic diversity and facilitate genetic rescue (i.e., mixing frogs from different populations) could have the opposite effect if gametes are locally adapted and the sperm of immigrants cannot function optimally in the new osmotic environment. This could present a barrier to fertilization and potentially exacerbate population declines (for an example immigrant reproductive dysfunction linked to sperm adaptation see Svensson et al. (2017)).

More broadly our findings contribute to our understanding of the evolution and diversification of sperm function by natural selection in freshwater external fertilizers. While past studies have demonstrated both phenotypic plasticity and local adaptation in sperm‐motility activation in euryhaline and saltwater external fertilizers (Green et al., 2020; Green, Niemax, et al., 2021; Svensson et al., 2017), similar studies are yet to be conducted for freshwater taxa. This is despite a rapidly growing literature investigating adaptive responses of freshwater organisms to salinization (Albecker et al., 2021; Albecker & McCoy, 2017). Combined with the findings of our previous study, results from the present study point towards local adaptation of sperm activation under freshwater conditions. Whether other freshwater external fertilizers show similar adaptations and whether adaptive sperm phenotypic plasticity might also contribute to the diversification of sperm function are questions that now require urgent attention. Evidence for species‐specific optima of sperm‐motility activation at fertilization medium osmolalities that match those at natural breeding sites in some frog and fish species (Morita et al., 2011; Rudin‐Bitterli et al., 2020) suggests that local adaptation in sperm physiology might be widespread. However, because freshwater systems can also experience high variation in salinity on temporal scales, we predict that sperm plasticity might be common among species that breed in highly variable osmotic environments. Freshwater taxa may also display some highly unusual evolutionary responses to challenging osmotic environments. For example, it was recently reported that a fish of freshwater ancestry is capable of marine spawning because it has evolved pre‐ejaculatory sperm activation that enables fertilization in the few seconds before sperm are immobilized by seawater (Beirao et al., 2018). Expanding our knowledge of sperm functioning in freshwater external fertilizers that actively breed in variable osmotic environments stands to significantly increase our understanding of the adaptive potential of sperm. This knowledge will be especially valuable given the acceleration of freshwater salinization globally and the increasing need to predict ecological and evolutionary responses to environmental change.

## AUTHOR CONTRIBUTIONS

**Phillip G. Byrne:** Conceptualization (equal); data curation (equal); formal analysis (equal); funding acquisition (equal); investigation (equal); methodology (equal); project administration (equal); resources (equal); supervision (equal); validation (equal); visualization (equal); writing – original draft (lead). **Zara M. Anastas:** Data curation (equal); investigation (equal); methodology (equal); writing – review and editing (equal). **Aimee J. Silla:** Conceptualization (equal); data curation (equal); funding acquisition (equal); investigation (equal); methodology (equal); project administration (equal); resources (equal); supervision (equal); validation (equal); visualization (equal); writing – review and editing (equal).

## CONFLICT OF INTEREST

The authors declare that they have no conflict of interest.

## DATA AVAILABILITY STATEMENT

Data are available on the Dryad Digital Repository https://doi.org/10.5061/dryad.w6m905qsj.

## Supporting information


Appendix S1
