# Supplementary material for: A test for plasticity in sperm motility activation in response to osmotic environment in an anuran amphibian
Source: Ecol Evol. 2022 Oct 1;12(10):e9387. doi: 10.1002/ece3.9387 (PMC9526115; doi:10.1002/ece3.9387)
Supplement: Supplementary file 1 — Appendix S1 [file ECE3-12-e9387-s001.pdf]

Appendix 1. Mean ± standard error mean (untransformed) for each sperm performance variable and acclimation treatment (Low = 0 mOsmolkg^{-1}; High = 50 mOsmolkg^{-1}) across six activation medium osmolalities.

	**Motility**		**VAP**		**VCL**	
**Activation osmolality**	**Low**	**High**	**Low**	**High**	**Low**	**High**
0	68.85 ± 2.27	71.71 ± 4.26	10.68 ± 0.23	10.92 ± 0.33	20.08 ± 0.64	20.28 ± 0.62
25	59.74 ± 4.45	62.25 ± 4.82	10.14 ± 0.37	10.95 ± 0.28	19.46 ± 0.97	21.77 ± 0.58
50	47.43 ± 3.82	39.29 ± 4.12	10.20 ± 0.17	10.98 ± 0.32	21.29 ± 0.53	22.59 ± 0.85
75	29.78 ± 3.13.	31.20 ± 4.59	10.32 ± 0.32	10.50 ± 0.36	21.73 ± 1.02	22.29 ± 0.69
100	19.32 ± 4.00	17.05 ± 2.76	9.22 ± 0.58	9.10 ± 0.65	19.26 ± 1.39	18.16 ± 1.31
200	14.08 ± 4.19	26.54 ± 5.86	7.75 ± 0.64	8.73 ± 0.55	15.37 ± 1.09	17.44 ± 1.34
